# Supplementary material for: Simple 3D Pose Features Support Human and Machine Social Scene Understanding
Source: ArXiv. 2026 Feb 19:arXiv:2511.03988v2. Preprint. [Version 2] (PMC12934831)
Supplement: Supplement 1 [file NIHPP2511.03988v2-supplement-1.pdf]

Supplemental materials

3D social pose features capture most social information from full 3D body joints

To determine whether our concise 3D social pose features (the combination of 3D positions and 3D directions) captured all the socially relevant information contained within the full body 3D body joints, we performed a semi-partial correlation analysis (see Methods). This analysis tested whether the full 3D joints contained any unique predictive information beyond what was already captured by our feature sets.

We first considered the two components of our social pose features, 3D positions and directions, separately. When partialling out only the 3D positions (Figure S1), the semi-partial correlations for spatial expanse (r = 0.09) and interagent distance (r = 0.20) show a clear decrease, but the correlation for agents facing (r = 0.75), communicative interaction (r = 0.28), and physical interaction (r = 0.32) are only minimally impacted. When partialling out only the 3D directions (Figure S1), the semi-partial correlation did not change across all dimensions (r = 0.798 for spatial expanse, r = 0.634 for interagent distance, r = 0.682 for agents facing, r = 0.437 for communicative interaction, and r = 0.458 for physical interaction). The remaining predictive power in both residualized conditions demonstrated that neither 3D positions nor 3D directions alone was sufficient to capture all socially relevant information from the joints.

However, when the complete 3D social pose features (combining both 3D positions and 3D directions) were partialled out from the 3D body joints, the unique predictive power of the remaining joint information showed dramatic drops across all five human ratings (r = 0.070 for spatial expanse, r = 0.241 for interagent distance, r = 0.081 for agents facing, r = 0.035 for communicative interaction, and r = 0.051 for physical interaction).

This result demonstrates that our concise 3D social pose features are both necessary (as individual components were insufficient) and largely sufficient (as the combined features left little remaining variance to be explained). The full, high-dimensional 3D joint data contains minimal unique, linearly decodable information relevant to these ratings beyond what is already captured by our combined 3D positions and 3D directions.

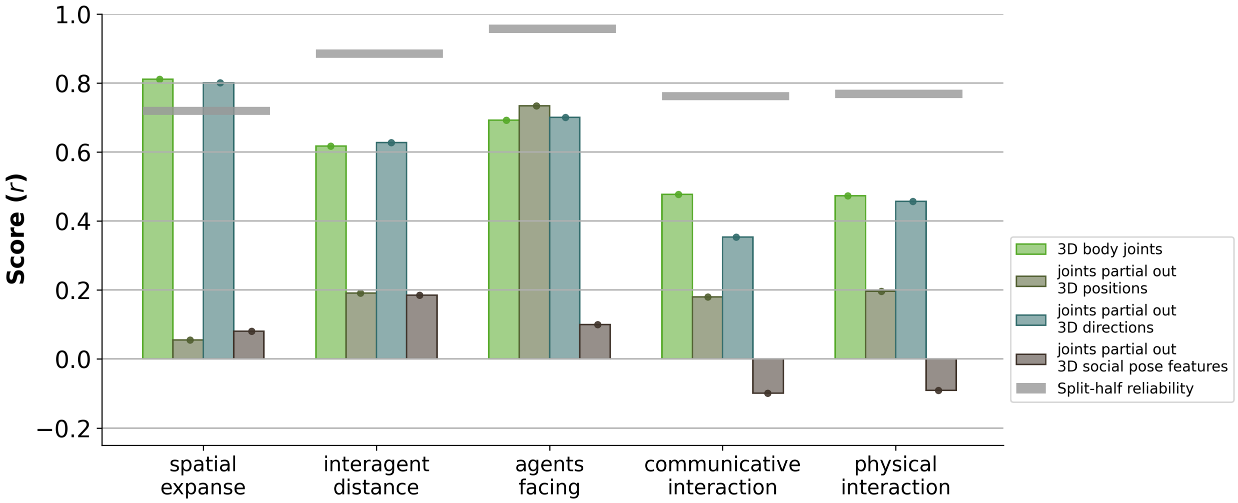

Figure S1. 3D social pose features capture most social information from 3D body joints. The bar chart displays the predictive performance (Pearson's *r*) for the full 3D body joints (light green) and the semi-partial correlation for residualized joint features, partialling out 3D positions (olive), 3D directions (teal), and the combination (dark brown). The dramatic drop in performance when the combination of position and direction is partialled out indicates that the full body 3D body joints contain little additional unique social information beyond the compact combination of 3D social pose features. Grey horizontal bars indicate the split-half reliability.

List of vision DNNs tested

model name	model type	spatial expanse	interagent distance	agents facing	communicative interaction	physical interaction
clip_vitl14	image	0.8089	0.7176	0.7763	0.6314	0.3346
clip_rn50x4	image	0.8278	0.6717	0.6973	0.53	0.5216
clip_vitb32	image	0.7556	0.6878	0.7621	0.5419	0.4581
clip_rn101	image	0.7848	0.6831	0.5918	0.5188	0.447
timm_convnext_large_in22ft1k	image	0.7506	0.6505	0.6609	0.6724	0.2791
timm_convnext_large	image	0.7506	0.6505	0.6609	0.6724	0.2791
timm_deit3_base_patch16_384_in21ft1k	image	0.8289	0.6075	0.6056	0.576	0.3739
timm_beitv2_base_patch16_224	image	0.7861	0.6293	0.701	0.4882	0.3759
timm_convnext_small	image	0.6986	0.5897	0.5889	0.5519	0.4594
timm_deit3_base_patch16_224_in21ft1k	image	0.8299	0.6927	0.6113	0.4775	0.2741
timm_deit_small_distilled_patch16_224	image	0.7813	0.7205	0.5627	0.41	0.4066
timm_deit3_large_patch16_384_in21ft1k	image	0.7746	0.6521	0.6135	0.5325	0.3066
torchvision_resnet50_imagenet1k_v2	image	0.7135	0.6175	0.6445	0.5489	0.3533
timm_convnext_large_in22k	image	0.7501	0.6328	0.6644	0.6114	0.2165
timm_gernet_m	image	0.7645	0.5981	0.6111	0.5571	0.3378
timm_beit_large_patch16_384	image	0.8143	0.7021	0.6121	0.3498	0.3796
timm_deit3_huge_patch14_224_in21ft1k	image	0.7827	0.6467	0.5848	0.5473	0.2952
timm_convnext_base_in22ft1k	image	0.8104	0.6588	0.3108	0.4839	0.5809
timm_convnext_base	image	0.8104	0.6588	0.3108	0.4839	0.5809
clip_vitb16	image	0.7587	0.6338	0.6726	0.4076	0.3687
timm_deit3_small_patch16_224_in21ft1k	image	0.7277	0.6177	0.6197	0.4813	0.3928
timm_lambda_resnet26t	image	0.7114	0.683	0.5983	0.4834	0.3542
torchvision_regnet_x_1_6gf_imagenet1k_v1	image	0.7869	0.566	0.5204	0.4735	0.4755
timm_dm_nfnet_f1	image	0.7363	0.6204	0.5701	0.574	0.2659

timm_edgenext_x_small	image	0.7984	0.6312	0.5029	0.4619	0.3692
torchvision_mnasnet1_0_imagenet1k_v1	image	0.7934	0.6026	0.5335	0.5162	0.3025
torchvision_regnet_x_800mf_imagenet1k_v1	image	0.7896	0.55	0.5769	0.5841	0.244
timm_deit3_small_patch16_384_in21ft1k	image	0.7346	0.6012	0.5988	0.4192	0.3879
timm_convnext_base_384_in22ft1k	image	0.7988	0.66	0.5962	0.2993	0.3829
timm_convnext_base_in22k	image	0.7566	0.5999	0.5026	0.4441	0.4168
timm_beit_base_patch16_224	image	0.8288	0.6409	0.6313	0.4762	0.1413
timm_deit3_large_patch16_384	image	0.7725	0.6467	0.5097	0.3796	0.4029
timm_legacy_seresnet50	image	0.8022	0.6505	0.5472	0.429	0.2741
slip_vit_b_yfcc15m	image	0.7981	0.6605	0.4841	0.4626	0.2939
x3d_xs	video	0.7218	0.7015	0.5197	0.3259	0.4216
timm_deit3_large_patch16_224	image	0.7904	0.5462	0.5178	0.5053	0.3288
timm_cait_xxs36_224	image	0.8049	0.6501	0.4652	0.3268	0.4379
timm_mixer_b16_224_miil	image	0.7836	0.5438	0.6205	0.4051	0.328
vicreg_resnet50_alpha0p75	image	0.7549	0.6162	0.3211	0.4895	0.499
timm_edgenext_base	image	0.7866	0.5993	0.5952	0.421	0.2736
timm_legacy_seresnext50_32x4d	image	0.7743	0.6547	0.4625	0.4463	0.3369
timm_gluon_resnet101_v1c	image	0.7346	0.6114	0.4201	0.5712	0.3359
timm_convnext_tiny	image	0.7955	0.6456	0.5452	0.4651	0.2208
timm_cait_s24_224	image	0.8094	0.6792	0.4569	0.4174	0.308
timm_convnext_pico	image	0.7134	0.6322	0.4344	0.4474	0.443
timm_jx_nest_tiny	image	0.7954	0.6187	0.5137	0.4462	0.2963
slip_vit_s_clip_yfcc15m	image	0.7157	0.5926	0.3954	0.5498	0.4115
torchvision_regnet_x_1_6gf_imagenet1k_v2	image	0.6936	0.633	0.5095	0.5539	0.275
dino_resnet50	image	0.7369	0.5882	0.5626	0.5381	0.2334
vissl_resnet50_pirl	image	0.801	0.5931	0.596	0.5421	0.1242
vissl_resnet50_swav	image	0.801	0.5931	0.596	0.5421	0.1242
vissl_resnet50_simclr	image	0.801	0.5931	0.596	0.5421	0.1242
timm_deit3_base_patch16_224	image	0.7711	0.5306	0.5734	0.4267	0.3539
slowfast_r50	video	0.6455	0.5847	0.5685	0.5601	0.2969
timm_convnext_small_in22k	image	0.8114	0.5661	0.4246	0.4373	0.4144
torchvision_swin_b_imagenet1k_v1	image	0.7516	0.5957	0.4419	0.4699	0.3912
timm_gluon_resnext101_32x4d	image	0.7298	0.6237	0.5137	0.5183	0.2619
timm_gernet_s	image	0.8253	0.6425	0.3869	0.4576	0.3211
torchvision_convnext_small_imagenet1k_v1	image	0.7602	0.5442	0.4368	0.5032	0.3876
torchvision_convnext_large_imagenet1k_v1	image	0.7316	0.6965	0.3301	0.4409	0.4271
timm_mobilenetv3_large_100_miil	image	0.7891	0.4879	0.6083	0.3781	0.3557
timm_beitv2_large_patch16_224	image	0.7984	0.6597	0.6625	0.2026	0.2928
x3d_s	video	0.7835	0.6258	0.5224	0.4451	0.2387
timm_convnext_nano	image	0.805	0.6324	0.5833	0.4811	0.1107
timm_eca_nfnet_l1	image	0.8009	0.5059	0.4996	0.2978	0.5038
timesformer-base-finetuned-k400	video	0.739	0.5617	0.5821	0.4329	0.2909
timm_convmixer_768_32	image	0.7756	0.6249	0.4804	0.3753	0.3492
timm_gluon_resnet152_v1c	image	0.7023	0.6442	0.4424	0.4935	0.3163
timm_cs3darknet_l	image	0.719	0.5842	0.4984	0.429	0.3679
torchvision_efficientnet_v2_s_imagenet1k_v1	image	0.7638	0.5872	0.4948	0.47	0.2815
timm_cs3darknet_m	image	0.7357	0.622	0.6353	0.4462	0.1573
dino_vitb8	image	0.811	0.6484	0.5523	0.3987	0.1805
torchvision_regnet_y_3_2gf_imagenet1k_v2	image	0.7257	0.6313	0.4021	0.4428	0.389
timm_cait_xs24_384	image	0.7608	0.6086	0.5349	0.464	0.2209
dino_xcit_medium_24_p16	image	0.8137	0.6187	0.5338	0.4706	0.1516
bit_expert_food	image	0.7766	0.5994	0.5448	0.5146	0.151
timm_gmlp_s16_224	image	0.7726	0.6225	0.4164	0.4129	0.362
timm_deit3_huge_patch14_224	image	0.7706	0.6328	0.4031	0.5308	0.249
timm_lamhalobotnet50ts_256	image	0.7282	0.6574	0.4391	0.463	0.2965
torchvision_regnet_y_3_2gf_imagenet1k_v1	image	0.7731	0.5932	0.3716	0.415	0.429
timm_mobilevitv2_150_384_in22ft1k	image	0.7315	0.591	0.5826	0.4657	0.2109
dino_vits16	image	0.7961	0.5373	0.6418	0.4192	0.184
torchvision_efficientnet_b1_imagenet1k_v2	image	0.7672	0.5114	0.537	0.3549	0.3995
timm_convit_base	image	0.7896	0.6389	0.4944	0.3567	0.2901
bit_expert_relation	image	0.764	0.6015	0.5572	0.5148	0.1297
vicreg_resnet50	image	0.7953	0.5523	0.5098	0.399	0.3062
timm_convit_small	image	0.7852	0.5857	0.6304	0.4463	0.1143

torchvision_regnet_x_400mf_imagenet1k_v1	image	0.6876	0.5266	0.5064	0.5594	0.2792
timm_dm_nfnet_f0	image	0.8287	0.5191	0.5027	0.5117	0.1956
timm_halonet50ts	image	0.7707	0.5389	0.4613	0.4639	0.3208
torchvision_convnext_base_imagenet1k_v1	image	0.8279	0.6266	0.5003	0.2199	0.3762
torchvision_swin_t_imagenet1k_v1	image	0.7804	0.6604	0.3223	0.4351	0.3526
timm_deit3_medium_patch16_224	image	0.7488	0.5817	0.5366	0.4454	0.2373
timm_ecaresnetlight	image	0.8124	0.6348	0.5852	0.3955	0.1209
torchvision_regnet_x_8gf_imagenet1k_v1	image	0.8024	0.5685	0.526	0.4123	0.2393
timm_cs3sedarknet_x	image	0.7975	0.5852	0.3159	0.4737	0.3744
timm_cspresnext50	image	0.7068	0.5961	0.4828	0.5247	0.2316
timm_cspresnet50	image	0.7698	0.5932	0.4088	0.4544	0.2995
vicreg_resnet50_alpha0p9	image	0.7358	0.6036	0.5365	0.382	0.2663
timm_deit3_large_patch16_224_in21ft1k	image	0.8022	0.5821	0.5864	0.2696	0.2771
timm_mobilenetv2_120d	image	0.7505	0.6374	0.5906	0.3917	0.1429
timm_dm_nfnet_f3	image	0.7881	0.4862	0.4283	0.4692	0.3379
timm_dla60	image	0.8025	0.6242	0.417	0.4869	0.179
torchvision_efficientnet_b1_imagenet1k_v1	image	0.7518	0.6215	0.4872	0.4361	0.21
timm_legacy_seresnet101	image	0.8245	0.6235	0.4964	0.3211	0.2388
timm_deit3_medium_patch16_224_in21ft1k	image	0.7849	0.6183	0.4603	0.3502	0.2904
timm_dpn92	image	0.7813	0.5733	0.3913	0.4452	0.3102
timm_legacy_senet154	image	0.6804	0.5652	0.5131	0.4324	0.3077
timm_levit_384	image	0.804	0.5437	0.5092	0.3984	0.2429
timm_convnext_nano_ols	image	0.7351	0.619	0.4787	0.4321	0.2315
bit_expert_arthropod	image	0.7717	0.615	0.434	0.4757	0.1959
i3d_r50	video	0.6893	0.5585	0.3042	0.6122	0.3279
bit_expert_flower	image	0.7802	0.6004	0.4257	0.5264	0.1584
timm_legacy_seresnet18	image	0.7546	0.5449	0.4428	0.4646	0.2823
timm_gluon_inception_v3	image	0.7515	0.6034	0.4997	0.5016	0.1312
slow_r50	video	0.7027	0.6435	0.4826	0.5279	0.1236
timm_deit_tiny_distilled_patch16_224	image	0.7753	0.5786	0.4914	0.4268	0.2073
timm_gluon_resnet50_v1b	image	0.7259	0.5374	0.5104	0.4234	0.2784
timm_edgenext_small	image	0.797	0.5299	0.4362	0.5003	0.2084
slip_vit_s_yfcc15m	image	0.7352	0.6241	0.3496	0.5126	0.25
timm_dla102x2	image	0.726	0.6423	0.3813	0.3583	0.3627
timm_gluon_resnext50_32x4d	image	0.6777	0.6435	0.453	0.4427	0.2538
dino_xcit_small_12_p16	image	0.8256	0.5962	0.5035	0.391	0.1526
timm_cait_xxs24_224	image	0.7872	0.5824	0.5531	0.3378	0.2062
timm_levit_256	image	0.7598	0.6334	0.5589	0.3601	0.146
timm_convnext_xlarge_in22k	image	0.7815	0.6701	0.4171	0.4488	0.1378
bit_expert_bird	image	0.7765	0.6459	0.4431	0.4551	0.1335
timm_cs3edgenet_x	image	0.7224	0.6015	0.418	0.4485	0.2636
clip_rn50	image	0.7098	0.6424	0.6778	0.431	-0.0079
bit_expert_instrument	image	0.7695	0.6229	0.441	0.4761	0.1432
timm_beit_large_patch16_224	image	0.8185	0.6405	0.525	0.4175	0.0502
timm_botnet26t_256	image	0.6959	0.6764	0.5671	0.4458	0.0631
timm_gluon_senet154	image	0.7297	0.5644	0.4607	0.436	0.2565
dino_xcit_medium_24_p8	image	0.8166	0.654	0.5092	0.2706	0.1966
timm_convnext_xlarge_in22ft1k	image	0.7742	0.676	0.4312	0.4302	0.1333
torchvision_swin_s_imagenet1k_v1	image	0.7827	0.5631	0.4251	0.3796	0.2943
timm_dla60x	image	0.7421	0.4712	0.4524	0.4172	0.3593
timm_deit_small_patch16_224	image	0.7357	0.5909	0.4591	0.4277	0.2252
timm_gluon_seresnext50_32x4d	image	0.7044	0.6263	0.4606	0.334	0.312
timm_halo2botnet50ts_256	image	0.7638	0.5997	0.5166	0.5417	0.0141
timm_mobilevit_s	image	0.7391	0.5147	0.6189	0.384	0.1762
torchvision_regnet_x_800mf_imagenet1k_v2	image	0.7427	0.4815	0.5756	0.5719	0.0611
timm_convmixer_1024_20_ks9_p14	image	0.7908	0.6078	0.4034	0.4062	0.2243
timm_gluon_resnext101_64x4d	image	0.7593	0.6274	0.4454	0.2864	0.3139
torchvision_regnet_y_800mf_imagenet1k_v1	image	0.7351	0.537	0.3739	0.4618	0.3248
torchvision_regnet_y_1_6gf_imagenet1k_v2	image	0.8032	0.4925	0.44	0.5224	0.1731
torchvision_mobilenet_v3_large_imagenet1k_v1	image	0.8015	0.4971	0.3946	0.4185	0.3159
torchvision_mnasnet1_3_imagenet1k_v1	image	0.6932	0.5376	0.5014	0.4913	0.2035
timm_ecaresnet50d	image	0.8182	0.4872	0.3948	0.4565	0.269
timm_beit_base_patch16_384	image	0.7786	0.6621	0.5813	0.2299	0.1724

timm_dla46x_c	image	0.7421	0.5753	0.4705	0.4093	0.2252
timm_deit_base_distilled_patch16_224	image	0.8032	0.4689	0.5316	0.3953	0.2227
timm_mixer_b16_224_miil_in21k	image	0.8015	0.554	0.3198	0.4304	0.3159
torchvision_resnext101_32x8d_imagenet1k_v2	image	0.7092	0.6704	0.3667	0.5083	0.1634
timm_convit_tiny	image	0.7504	0.5993	0.5701	0.3566	0.1416
bit_expert_object	image	0.7715	0.6122	0.439	0.465	0.1277
timm_convnext_tiny_in22ft1k	image	0.7617	0.565	0.4912	0.4384	0.1574
torchvision_mobilenet_v3_small_imagenet1k_v1	image	0.7403	0.5131	0.4941	0.3639	0.3017
vissl_resnet50_deepclusterv2	image	0.7388	0.5837	0.4622	0.5423	0.086
timm_deit3_small_patch16_224	image	0.7807	0.4951	0.5107	0.5225	0.1019
timm_cs3darknet_focus_l	image	0.7019	0.5189	0.5037	0.4309	0.2546
torchvision_regnet_y_16gf_imagenet1k_v1	image	0.7523	0.4894	0.4866	0.4109	0.2683
timm_mixer_b16_224	image	0.7497	0.5962	0.3957	0.3842	0.2809
timm_mixer_b16_224_in21k	image	0.7497	0.5962	0.3957	0.3842	0.2809
timm_gluon_resnet50_v1d	image	0.8017	0.5135	0.359	0.3691	0.3626
torchvision_regnet_y_32gf_imagenet1k_v2	image	0.7402	0.5201	0.4323	0.3822	0.3308
timm_dla102x	image	0.7483	0.5859	0.475	0.4287	0.1656
timm_cs3darknet_x	image	0.7487	0.5354	0.5086	0.4163	0.1925
torchvision_swin_v2_b_imagenet1k_v1	image	0.7535	0.5072	0.4343	0.3197	0.3837
timm_levit_192	image	0.7742	0.4129	0.4963	0.546	0.1684
torchvision_regnet_y_400mf_imagenet1k_v2	image	0.7348	0.4765	0.5207	0.3067	0.3581
torchvision_shufflenet_v2_x1_5_imagenet1k_v1	image	0.7934	0.4378	0.5218	0.4207	0.2227
timm_jx_nest_base	image	0.7851	0.5698	0.4392	0.3652	0.237
timm_mobilevitv2_150	image	0.7171	0.582	0.5419	0.3832	0.1668
bit_expert_vehicle	image	0.7666	0.6086	0.4512	0.4307	0.1335
slip_vit_s_simclr_yfcc15m	image	0.7746	0.5456	0.3739	0.4481	0.2473
timm_cspdarknet53	image	0.6817	0.5432	0.445	0.5563	0.1621
timm_densenet121	image	0.8072	0.5385	0.509	0.356	0.1766
bit_expert_abstraction	image	0.7647	0.614	0.4403	0.4436	0.121
torchvision_resnet101_imagenet1k_v1	image	0.7571	0.6102	0.3132	0.5384	0.1634
timm_gmixer_24_224	image	0.771	0.567	0.3812	0.4322	0.2306
timm_halonet26t	image	0.753	0.6144	0.5976	0.3358	0.0811
torchvision_resnet34_imagenet1k_v1	image	0.7962	0.5937	0.4995	0.2635	0.2289
torchvision_mobilenet_v2_imagenet1k_v2	image	0.7382	0.5901	0.3623	0.5305	0.1606
timm_efficientformer_l1	image	0.7476	0.5033	0.3834	0.4063	0.3403
timm_gluon_resnet50_v1c	image	0.7392	0.5382	0.528	0.4904	0.0839
timm_deit_base_distilled_patch16_384	image	0.7271	0.5959	0.4603	0.4143	0.181
timm_hardcorenas_a	image	0.7675	0.5539	0.5522	0.3712	0.1308
torchvision_regnet_y_800mf_imagenet1k_v2	image	0.7578	0.5793	0.4173	0.4179	0.2016
timm_hardcorenas_f	image	0.7561	0.5292	0.477	0.3734	0.2358
timm_cait_xxs36_384	image	0.7361	0.6453	0.5179	0.2735	0.1952
torchvision_alexnet_imagenet1k_v1	image	0.7293	0.4987	0.3443	0.429	0.3623
x3d_m	video	0.715	0.5581	0.4617	0.4364	0.1922
timm_mobilenetv3_small_075	image	0.7023	0.4729	0.4749	0.4413	0.2716
timm_deit3_small_patch16_384	image	0.7528	0.6041	0.4397	0.46	0.1036
timm_mnasnet_100	image	0.7555	0.4988	0.479	0.3969	0.2281
torchvision_regnet_x_3_2gf_imagenet1k_v2	image	0.649	0.6495	0.4706	0.5223	0.064
timm_dm_nfnet_f2	image	0.7816	0.6535	0.6721	0.3632	-0.1157
torchvision_mnasnet0_75_imagenet1k_v1	image	0.6924	0.5537	0.3551	0.4937	0.259
timm_convnext_tiny_in22k	image	0.7599	0.5338	0.3997	0.4497	0.2056
torchvision_resnext50_32x4d_imagenet1k_v2	image	0.7338	0.6331	0.5033	0.43	0.048
bit_expert_mammal	image	0.7628	0.6143	0.4818	0.3028	0.1853
timm_convnext_atto	image	0.7573	0.5465	0.5479	0.425	0.0652
vissl_resnet50_jigsaw_p100	image	0.7226	0.6083	0.374	0.4301	0.2047
timm_gluon_resnet34_v1b	image	0.7354	0.5529	0.4339	0.4282	0.1864
torchvision_regnet_y_16gf_imagenet1k_v2	image	0.8025	0.5746	0.3672	0.4175	0.174
vissl_resnet50_supervised	image	0.7944	0.5608	0.3421	0.431	0.2062
torchvision_shufflenet_v2_x1_0_imagenet1k_v1	image	0.7659	0.4924	0.3692	0.5145	0.1909
timm_deit3_base_patch16_384	image	0.7928	0.5785	0.2467	0.4431	0.2715
timm_dla169	image	0.688	0.5638	0.4199	0.3751	0.282
timm_mobilenetv3_small_050	image	0.7077	0.4937	0.4526	0.511	0.1627
timm_efficientformer_l7	image	0.7932	0.5933	0.3583	0.388	0.1926
timm_mobilenetv3_large_100	image	0.7698	0.5315	0.422	0.5448	0.0573

bit_expert_animal	image	0.7687	0.6204	0.4648	0.3269	0.1436
timm_deit_base_patch16_384	image	0.6968	0.532	0.4264	0.4596	0.2034
timm_gluon_resnet101_v1s	image	0.7556	0.6594	0.3772	0.5938	-0.0705
timm_convnext_tiny_hnf	image	0.7371	0.5301	0.5311	0.4448	0.0722
torchvision_ssd300_vgg16_coco_v1	image	0.8172	0.6585	0.4968	0.122	0.2197
timm_mobilenetv2_050	image	0.7122	0.4812	0.3993	0.5127	0.2025
dino_vits8	image	0.6414	0.5855	0.4134	0.4441	0.2226
timm_levit_128	image	0.7982	0.4992	0.4664	0.4187	0.1214
timm_deit_base_patch16_224	image	0.7244	0.5369	0.4038	0.4223	0.2161
slip_vit_b_simclr_yfcc15m	image	0.7786	0.5197	0.5082	0.2918	0.2026
torchvision_convnext_tiny_imagenet1k_v1	image	0.793	0.6057	0.3446	0.4262	0.1302
timm_convnext_tiny_384_in22ft1k	image	0.7556	0.5602	0.4162	0.3542	0.2131
torchvision_shufflenet_v2_x0_5_imagenet1k_v1	image	0.7532	0.51	0.2573	0.5074	0.2703
torchvision_mobilenet_v3_large_imagenet1k_v2	image	0.7494	0.5056	0.4267	0.3853	0.2285
timm_inception_v4	image	0.7506	0.479	0.5377	0.3845	0.1431
torchvision_regnet_x_400mf_imagenet1k_v2	image	0.7256	0.5221	0.4122	0.336	0.2958
timm_hardcorenas_c	image	0.7854	0.4972	0.5458	0.3311	0.1299
timm_edgenext_small_rw	image	0.7994	0.5077	0.5089	0.3336	0.1391
timm_mobilenetv2_110d	image	0.7664	0.4854	0.34	0.4399	0.2471
timm_haloregnetz_b	image	0.7415	0.5096	0.4023	0.3559	0.2676
timm_mobilevitv2_050	image	0.7624	0.555	0.4813	0.3386	0.1395
torchvision_resnet18_imagenet1k_v1	image	0.7803	0.543	0.4728	0.3866	0.0905
timm_gluon_resnet101_v1b	image	0.7229	0.6223	0.5207	0.4298	-0.0225
vissl_resnet50_mocov2	image	0.7933	0.5943	0.3905	0.4162	0.0716
timm_mobilenetv2_100	image	0.7642	0.5666	0.4456	0.3325	0.1563
timm_gluon_resnet152_v1b	image	0.7013	0.6136	0.3492	0.4133	0.1843
timm_cait_s24_384	image	0.7051	0.6052	0.3252	0.3104	0.3116
timm_bat_resnext26ts	image	0.7417	0.5857	0.16	0.4581	0.3119
timm_convmixer_1536_20	image	0.7604	0.4944	0.4794	0.397	0.1191
torchvision_resnext101_64x4d_imagenet1k_v1	image	0.6604	0.5875	0.4834	0.2365	0.2819
slip_vit_b_clip_yfcc15m	image	0.7518	0.6038	0.3355	0.4	0.1579
timm_cait_s36_384	image	0.7633	0.6635	0.4221	0.3462	0.0521
timm_convnext_small_in22ft1k	image	0.7637	0.5395	0.4682	0.3447	0.13
timm_ecaresnet50t	image	0.7832	0.4778	0.4326	0.4841	0.0678
timm_hardcorenas_e	image	0.7407	0.5263	0.4266	0.2954	0.256
timm_efficientnet_b0	image	0.7885	0.5128	0.539	0.2505	0.1517
torchvision_resnext50_32x4d_imagenet1k_v1	image	0.8236	0.6071	0.1015	0.4661	0.2406
timm_mobilevit_xxs	image	0.8066	0.5449	0.4466	0.3296	0.1093
timm_mobilevit_xs	image	0.7874	0.5761	0.4698	0.2662	0.1372
timm_eca_resnet33ts	image	0.7545	0.5112	0.493	0.2616	0.2144
timm_mobilenetv3_large_100_miil_in21k	image	0.771	0.5731	0.4246	0.4785	-0.0131
torchvision_resnet152_imagenet1k_v1	image	0.801	0.4099	0.4077	0.1697	0.4442
torchvision_shufflenet_v2_x2_0_imagenet1k_v1	image	0.7131	0.5295	0.5435	0.5033	-0.0571
timm_gluon_resnet152_v1d	image	0.7135	0.6344	0.2567	0.5372	0.0901
timm_efficientnet_b2	image	0.7822	0.5356	0.4069	0.2725	0.2284
timm_inception_resnet_v2	image	0.7791	0.6238	0.4585	0.2774	0.0865
timm_ghostnet_100	image	0.7976	0.5858	0.5587	0.3405	-0.0625
torchvision_ssdlite320_mobilenet_v3_large_coco_v1	image	0.768	0.6451	0.272	0.4239	0.1107
timm_mobilevitv2_150_in22ft1k	image	0.731	0.477	0.3362	0.348	0.3272
timm_hardcorenas_d	image	0.7333	0.5149	0.4268	0.3583	0.1859
timm_convnext_pico_ols	image	0.7656	0.5545	0.4077	0.3986	0.0919
timm_ig_resnext101_32x32d	image	0.7226	0.5594	0.295	0.4756	0.1656
timm_ig_resnext101_32x16d	image	0.7226	0.5594	0.295	0.4756	0.1656
timm_ig_resnext101_32x8d	image	0.7226	0.5594	0.295	0.4756	0.1656
timm_ig_resnext101_32x48d	image	0.7226	0.5594	0.295	0.4756	0.1656
timm_cait_xxs24_384	image	0.7873	0.5839	0.5085	0.4054	-0.0681
timm_levit_128s	image	0.7554	0.5405	0.4035	0.4176	0.0976
timm_eca_resnext26ts	image	0.6497	0.5038	0.5106	0.4016	0.148
timm_mobilevitv2_075	image	0.7319	0.5445	0.5424	0.2557	0.1384
timm_densenet201	image	0.7133	0.5673	0.318	0.358	0.256
torchvision_resnet101_imagenet1k_v2	image	0.7214	0.56	0.4893	0.5135	-0.074
timm_mobilevitv2_125	image	0.7384	0.5104	0.4966	0.3304	0.1315
timm_dm_nfnet_f4	image	0.7683	0.4679	0.3572	0.4799	0.1328

timm_mixer_l16_224_in21k	image	0.7466	0.5074	0.3161	0.3135	0.3198
timm_mixer_l16_224	image	0.7466	0.5074	0.3161	0.3135	0.3198
torchvision_regnet_y_400mf_imagenet1k_v1	image	0.7413	0.4935	0.5198	0.3791	0.0667
timm_dla102	image	0.7092	0.6133	0.3882	0.3459	0.1408
torchvision_regnet_y_8gf_imagenet1k_v2	image	0.7012	0.4944	0.4507	0.4229	0.1281
timm_convnext_femto_ols	image	0.778	0.4716	0.3481	0.4967	0.0992
timm_convnext_atto_ols	image	0.7274	0.4354	0.5295	0.3632	0.1339
timm_dla46_c	image	0.8216	0.5016	0.4258	0.422	0.0136
timm_hardcorenas_b	image	0.7388	0.4791	0.5284	0.308	0.1228
timm_gluon_resnet101_v1d	image	0.6434	0.5673	0.2849	0.4331	0.2416
torchvision_regnet_x_16gf_imagenet1k_v2	image	0.7278	0.5014	0.4388	0.3486	0.1519
timm_gluon_resnet18_v1b	image	0.752	0.5442	0.3602	0.3535	0.1578
torchvision_regnet_x_3_2gf_imagenet1k_v1	image	0.7178	0.4522	0.3204	0.4663	0.2081
timm_lcnet_100	image	0.7827	0.536	0.4504	0.2069	0.1881
timm_mvitv2_base	image	0.7543	0.5704	0.5032	0.4639	-0.129
timm_inception_v3	image	0.7661	0.4786	0.2779	0.4788	0.1603
timm_mobilenetv3_rw	image	0.7691	0.5035	0.3319	0.4195	0.1361
torchvision_resnext101_32x8d_imagenet1k_v1	image	0.7762	0.5754	0.1969	0.3776	0.231
timm_ecaresnet26t	image	0.7326	0.5678	0.3292	0.4777	0.0486
timm_cs3darknet_focus_m	image	0.7961	0.5371	0.4674	0.4917	-0.1446
timm_legacy_seresnet34	image	0.7835	0.6036	0.3491	0.4289	-0.019
timm_densenet169	image	0.5511	0.5115	0.4031	0.4615	0.2091
timm_efficientnet_b1	image	0.6615	0.4573	0.4815	0.4198	0.1113
torchvision_mobilenet_v2_imagenet1k_v1	image	0.7786	0.4402	0.3555	0.426	0.1211
timm_lambda_resnet26rpt_256	image	0.7237	0.6085	0.2321	0.4881	0.0657
dino_vitb16	image	0.8148	0.548	0.0886	0.4409	0.2221
timm_ecaresnet269d	image	0.742	0.5016	0.5273	0.2732	0.069
torchvision_densenet169_imagenet1k_v1	image	0.5514	0.5263	0.3956	0.4376	0.2022
timm_dla34	image	0.7551	0.5184	0.4438	0.312	0.0829
timm_lcnet_075	image	0.7438	0.5293	0.3299	0.2675	0.2405
vissl_resnet50_clusterfit	image	0.7686	0.5662	0.2448	0.3935	0.1299
torchvision_densenet121_imagenet1k_v1	image	0.6889	0.5022	0.4292	0.358	0.1204
timm_mobilenetv2_140	image	0.7526	0.4949	0.2789	0.4348	0.1327
torchvision_regnet_y_8gf_imagenet1k_v1	image	0.7942	0.5928	0.3096	0.4036	-0.0094
timm_edgenext_xx_small	image	0.7944	0.5335	0.5147	0.1278	0.1205
timm_deit_tiny_patch16_224	image	0.7285	0.4965	0.4339	0.2595	0.1673
timm_darknet53	image	0.6459	0.6054	0.2323	0.4527	0.1483
timm_ecaresnet101d	image	0.836	0.533	0.3541	0.223	0.1377
timm_legacy_seresnext26_32x4d	image	0.7734	0.562	0.3535	0.3331	0.0612
timm_gluon_seresnext101_64x4d	image	0.8212	0.604	0.3698	0.5227	-0.2389
timm_gluon_seresnext101_32x4d	image	0.7768	0.4124	0.143	0.4789	0.263
timm_lcnet_050	image	0.6679	0.4597	0.3826	0.2207	0.3386
timm_dla60x_c	image	0.7786	0.5474	0.2899	0.2251	0.2277
timm_darknetaa53	image	0.7266	0.5523	0.2712	0.5034	0.0058
torchvision_mnasnet0_5_imagenet1k_v1	image	0.7479	0.4795	0.4128	0.4231	-0.0075
timm_mnasnet_small	image	0.6649	0.5395	0.607	0.0358	0.2076
vissl_resnet50_jigsaw_goyal19	image	0.7013	0.5397	0.3567	0.4384	0.0126
timm_mixnet_m	image	0.7943	0.4956	0.3089	0.2048	0.2423
vissl_resnet50_rotnet	image	0.701	0.5713	0.1629	0.3847	0.2184
timm_cs3sedarknet_l	image	0.7197	0.4533	0.4444	0.3519	0.0681
timm_convnext_femto	image	0.7797	0.4834	0.1975	0.3765	0.181
torchvision_resnet50_imagenet1k_v1	image	0.7322	0.477	0.28	0.3623	0.1555
dino_xcit_small_12_p8	image	0.8229	0.6776	0.4106	0.3279	-0.2367
timm_jx_nest_small	image	0.7662	0.6071	0.565	0.209	-0.1565
torchvision_regnet_y_32gf_imagenet1k_v1	image	0.7467	0.454	0.2998	0.1169	0.3332
torchvision_densenet201_imagenet1k_v1	image	0.7144	0.5664	0.3134	0.3475	0.006
torchvision_inception_v3_imagenet1k_v1	image	0.7665	0.4812	0.2544	0.2836	0.161
timm_eca_nfnet_l2	image	0.7934	0.3318	0.186	0.2123	0.4089
timm_lambda_resnet50ts	image	0.7441	0.6037	0.1522	0.3766	0.042
timm_gluon_resnet152_v1s	image	0.6594	0.4908	0.3589	0.3501	0.041
timm_mobilenetv3_small_100	image	0.732	0.574	0.4958	-0.0076	0.0777
timm_mobilevitv2_100	image	0.7801	0.5201	0.1054	0.3113	0.1447
timm_mixnet_s	image	0.7424	0.5093	0.3785	0.2781	-0.0468

timm_legacy_seresnext101_32x4d	image	0.6634	0.4777	0.3745	0.1255	0.2168
torchvision_squeezenet1_0_imagenet1k_v1	image	0.6452	0.4969	0.3821	0.171	0.1235
timm_legacy_seresnet152	image	0.812	0.6272	0.0837	0.0999	0.1531
timm_densenet161	image	0.666	0.4925	0.2929	0.3532	-0.0611
torchvision_squeezenet1_1_imagenet1k_v1	image	0.7709	0.429	0.2259	0.2374	0.0797
torchvision_resnet152_imagenet1k_v2	image	0.6981	0.572	0.2959	0.2435	-0.0836
timm_cs3se_edgenet_x	image	0.772	0.6367	0.0091	0.2194	0.0789
torchvision_regnet_x_8gf_imagenet1k_v2	image	0.6967	0.5006	0.3604	-0.0054	0.161
c2d_r50	video	0.6384	0.5905	0.1191	0.1476	0.1294
torchvision_regnet_y_1_6gf_imagenet1k_v1	image	0.7651	0.4819	0.429	0.2633	-0.3227
timm_efficientformer_l3	image	0.8125	0.5459	0.0514	-0.132	0.1477
torchvision_densenet161_imagenet1k_v1	image	0.6656	0.493	0.3019	-0.0464	-0.0809

Supplemental table 1: List of vision DNNs tested. This table provides a complete list of all pretrained image and video deep neural networks included in our analysis, ranked by their performance on the average score across five ratings. The naming convention is from DeepJuice (Conwell et al., 2024).
